# Do people rely more on habits when sleepy? An ecological momentary assessment study

**DOI:** 10.1111/jsr.14421

**Published:** 2024-12-15

**Authors:** Theresa McLaurin, Benjamin Gardner, Alexandra E. Shriane, Amanda L. Rebar, Grace E. Vincent

**Affiliations:** ^1^ Appleton Institute; School of Health, Medical and Applied Sciences Central Queensland University Rockhampton Queensland Australia; ^2^ Habit Application and Theory group, School of Psychology University of Surrey Guildford UK; ^3^ Motivation of Health Behaviors Lab; Health Promotion, Education, & Behavior; Arnold School of Public Health University of South Carolina Columbia South Carolina USA

**Keywords:** automaticity, daily behaviour, dual process, fatigue, momentary

## Abstract

When self‐regulatory resources are depleted, people tend to act more on “autopilot”, with minimal forethought. It follows that when sleepy, people should be more likely to act habitually, based on learned cue–behaviour associations that trigger behaviour automatically when the cue is encountered. This ecological momentary assessment study investigated whether, over the course of a week, between‐person differences and momentary within‐person variation in daytime sleepiness were associated with the reported habit strength of behaviours. Participants (*N* = 105, 71% female, M age = 35 years) completed a baseline assessment of sleep quality and, six times daily over 7 days, momentary assessments in which they reported the habit strength of the behaviour they were doing when prompted and their momentary sleepiness. Multilevel modelling revealed that people who were more sleepy *than others* were not more or less likely to act habitually, but on occasions when people were more sleepy *than was typical for them*, the behaviour they were engaging in tended to be more habitual (*Pseudo‐R* ^2^ = 2%). Our results suggest that sleepiness causes people to rely on non‐reflective processes such as habit to regulate their behaviour. Interventions should promote habit formation for desirable behaviours so that when people are sleepy, they can rely on the efficiency of habits to ensure they continue to enact wanted behaviours. Conversely, interventions promoting behaviours that require deliberative thought might encourage performance during times of day when people are more alert.

## 1 INTRODUCTION

Many people experience high levels of daytime sleepiness. The prevalence of sleep problems in the general population is increasing (Adams et al., 2017; D'Ambrosio et al., 2019), and 23% of American adults report being impacted by inadequate sleep during waking hours (Kolla et al., 2020). Daytime sleepiness is characterised by reduced wakefulness and alertness, and an increased propensity to fall asleep during typical waking hours (Berger et al., 2021). Much like hunger and thirst are detected when fasting, sleepiness is the body's warning signal of the need for sleep, which is generally felt after extended periods of wakefulness (Mairesse et al., 2014; Odle‐Dusseau et al., 2010).

Aside from being an unpleasant experience, one of the adverse consequences of sleepiness is that it renders people less able to consciously regulate their behaviour. At any given moment, people are subject to multiple desires that compete to guide their action. Acting in a purposeful, goal‐directed way typically requires diligent, attentive monitoring of behavioural responses and the momentary prioritisation of desires that best serve one's goals (Baumeister et al., 2000; Hofmann et al., 2009). The Controlled Attention Model proposes that sleep‐deprived individuals will have difficulty enacting tasks that require effortful attention (Pilcher et al., 2015). Evidence shows that sleep deprivation and poorer sleep quality lead to a decrease in self‐regulatory resources including self‐control (Guarana et al., 2021; Hagger, 2010; Pilcher et al., 2015). Diminished self‐regulatory capacity compromises the ability to effectively negotiate competing motivational forces, and may lead people to momentarily act contrary to their broader goals.

One psychological mechanism with the potential to counteract the impact of self‐regulatory losses on behaviour is habit. Habit can be defined as a psychological process whereby encountering a context activates a learned context–behaviour association, which in turn triggers an impulse to enact the associated behaviour (Gardner, 2015); “habitual behaviour” describes action generated by this process (Gardner & Lally, 2023). Habits are acquired through a process of context‐consistent repetition: consistently enacting a behaviour (e.g. having a beer) in a context (e.g. when returning home after work) reinforces a context–behaviour association, to the extent that merely encountering the context is sufficient to activate an unconscious impulse to initiate the behaviour (Lally et al., 2010; Wood, 2017). By virtue of its automatic activation, a habitual behaviour can proceed even when people are momentarily unable to muster the willpower to act (Gardner et al., 2020). Studies show that, while people are typically less able to engage in desired, non‐habitual behaviour when their self‐regulatory capacity is depleted, they can continue to enact habitual behaviours even when self‐regulation is diminished (Neal et al., 2013). Habit formation can thereby protect wanted behaviours from likely derailment in instances where people lack attention, motivation or self‐regulatory resources (de Ridder & Gillebaart, 2017). Habit theory would therefore predict that, when people are sleepy, and so less able to self‐regulate, they should report a greater reliance on automatic processes; that is, when sleepy, people should act more habitually. Yet, to our knowledge, no research has yet examined whether daytime sleepiness is associated with engaging in habitual behaviours.

## 2 THE PRESENT STUDY

Past research suggests that depletion of self‐regulatory capacity increases enactment of habitual behaviours (Neal et al., 2013), and that sleepiness weakens self‐regulatory capabilities (Guarana et al., 2021). This study aimed to bridge these two findings, by examining whether people who are sleepy report acting more habitually. We used an ecological momentary assessment design to investigate ongoing behaviours, how strongly habitual those behaviours were, and ratings of sleepiness.

We sought to model effects of sleepiness both between and within participants, because it has been proposed that sleepiness at any given time arises from a combination of a general sleepiness “trait”, and state‐level within‐person fluctuations (De Valck & Cluydts, 2003). General sleepiness refers to a base, person‐specific level of sleepiness that is constant across days and weeks (e.g. related to genetic factors), irrespective of situational variables. In contrast, state sleepiness reflects feelings of sleepiness that vary periodically, within individuals, as a result of changes in situational factors (e.g. exposure to light and the time of the day) or endogenous physiological processes (e.g. the circadian rhythm and sleep homeostasis; Bakotic & Radosevic‐Vidacek, 2013; Dijk & Archer, 2010). Our ecological momentary assessment design aimed to parse out both between‐person effects attributable to individual differences, and within‐person effects attributable to momentary fluctuations across the day (Shiffman et al., 2008). We predicted the following.

Hypothesis 1 People with higher overall daytime sleepiness will engage in more habitual behaviours than people with lower daytime sleepiness (between‐person effect).

Hypothesis 2 On occasions when people are sleepier than is typical for them, they will engage in more strongly habitual behaviours compared with when they are less sleepy (within‐person effect).

## 3 METHODS

### 3.1 Participants

Convenience sampling was used to recruit 105 adults from the general population in Australia and the UK using social media posts. Individuals were eligible to participate if they were aged 18 years or older, and had a mobile phone that could receive text messages throughout their day. There was sufficient power for medium‐sized within‐person associations (Amatya & Bhaumik, 2018), and the sample size was adequate to test the hypothesis regarding between‐person differences in overall daytime sleepiness and habit strength (Faul et al., 2007) based on data from Barber et al. that found a medium negative correlation between poor sleep hygiene and self‐control levels (*r* = −0.42; Barber et al., 2013).

### 3.2 Study design and procedure

Data were collected January 2022–August 2023. Participants reported their real‐time experiences in their natural context six times per day for 7 consecutive days. The recruitment advertisement included a link to an online baseline survey where informed consent, demographic data and general sleep quality were self‐reported (Qualtrics XM, 2005). Members of the research team then contacted participants via email and asked them to nominate six, 2‐hr windows of time that they would be available to respond to prompts for each day of the following week. A time randomisation Excel spreadsheet was used to generate the times that text messages would be sent each day of the week within each of the six, 2‐hr windows nominated by participants, with a total of 42 messages sent across the week (Microsoft Corporation, 2022).

The text messages contained a link to “prompt surveys”, which asked participants to “Please describe what you were doing when you were prompted” (open response) and rate how habitual that behaviour was and their current level of sleepiness. ClickSend (Clicksend, 2022) was used to schedule messages to participants from a dedicated number, and participants were given 30 min to respond before the responses were treated as missing. Australian participants received a $50 AUD (~$35 USD) electronic gift voucher upon completion of the study, and Australian participants who completed more than 85% of prompt surveys received an additional $10 AUD (~$6.50 USD) electronic gift voucher. UK participants received a £20 (~$25 USD) electronic gift voucher if they completed 50% of prompt surveys or a £30 (~$25 USD) electronic gift voucher if they completed 85% of prompt surveys. This study protocol was approved by the hosting institutions' Human Research Ethics Committees (Australia: Approval No. 23216; UK: LRU/DP‐21/22–26,918).

### 3.3 Measures

Demographic characteristics were measured in the baseline survey only, and included self‐reported age (in years), gender (*male, female, transgender, non‐binary, not listed here – please specify, prefer not to say*), highest level of education completed (Australia and UK options varied in line with country education systems), marital status (*single, married, widowed, divorced, separated, prefer not to say*) and main employment status (*working [part‐time employee]; working [full‐time employee]; working [self‐employed]; not working [looking for work]; not working due to disability; not working (other); retired; part‐time student; full‐time student; prefer not to answer*).

Sleep quality was assessed at baseline only. Participants completed the sleep quality sub‐scale from the Pittsburgh Sleep Quality Index (Buysse et al., 1989): “During the past month, how would you rate your sleep quality overall?” with the response options ranging from 1 (*very good*) to 4 (*very bad*). Responses from this item were reverse‐coded so that higher scores were indicative of better sleep quality.

Behavioural habit strength was assessed in the prompt surveys, using two items from the valid and reliable, four‐item Self‐Report Behavioural Automaticity Index (SRBAI; Gardner et al., 2012). The SRBAI provides an indication of the likely automatic nature of the influence of habit on behaviour. While concerns have been raised about the validity of self‐reporting habit (Hagger et al., 2015), the SRBAI reliably infers habit from participants' reflections on the “symptoms” of habitual responding, such as whether the behaviour tends to be initiated without conscious awareness or cognitive effort (Orbell & Verplanken, 2015). The SRBAI has been shown to have construct validity in that people generally understand the items (Gardner & Tang, 2014), and responses to those items perform as expected in predicting behaviour (Gardner et al., 2012; Rebar, Gardner, et al., 2018).

After reporting the behaviour they were engaged in when prompted, participants were asked to rate the extent to which that behaviour was habitually instigated (i.e. selected automatically, rather than mindfully; Gardner et al., 2012; Gardner et al., 2016): i.e. the decision to do the behaviour was something “I did automatically” and “I did without thinking”. Response options ranged from 1 (*strongly disagree*) to 7 (*strongly agree*). Item responses were averaged to calculate a total habit strength score, with higher scores indicative of more habitual behaviour. Two items were used to reduce participant burden as is common in repeated measures studies; the items selected reliably strongly loaded onto the automaticity factor of the four‐item index and demonstrated sufficient internal consistency in the present data (*α* = 0.90, Guttman's *λ* = 0.82).

Momentary sleepiness was measured in the prompt surveys, using the 10‐point version of the Karolinska Sleepiness Scale (Shahid et al., 2011), in which participants were asked to indicate how sleepy they felt (in that moment) using a scale ranging from 1 (*extremely alert*) to 10 (*extremely sleepy, can't keep awake*). This scale has been shown to be a valid measurement of situational stress, sensitive to fluctuations (Kaida et al., 2006; Putilov & Donskaya, 2013; Shahid et al., 2011).

### 3.4 Data management and analyses

Data analyses were conducted using *R* version 4.3.1 (R Core Team, 2023). All surveys completed within 30 min of the prompt were included in analyses. Missing responses within prompt surveys (< 0.01%) were imputed with the median. Intraclass correlations (ICCs) were calculated to establish the need to account for nesting of data within‐person across time. ICCs represent proportions of between‐ to within‐person variability, with a higher value indicating more between‐person variability relative to within‐person.

To test whether daytime sleepiness was positively associated with behavioural habit strength at the between‐ and within‐person levels, a linear mixed‐effect model was fit, which optimised restricted maximum likelihood criterion in the *lmer* function of the *lme4* package (Bates et al., 2015). Daytime sleepiness was parsed into within‐person and between‐person levels: between‐person level sleepiness was calculated as individuals' scores averaged across occasions, centred at the grand mean, and entered at Level 2 of the model, and within‐person fluctuations in sleepiness were calculated as occasion scores centred at the person's mean and entered at Level 1 in the model. Age, gender, cohort (Australia versus UK) and sleep quality were included as model covariates. Confidence intervals (CIs) estimated from 100 posterior simulations were used to test if the model coefficient estimates statistically significantly differed from zero. Prior to model estimation, data were visually inspected for outliers and violations of assumptions for the analyses with no abnormalities or unmet assumptions detected.

## 4 RESULTS

### 4.1 Sample characteristics and descriptive statistics

Of the 105 participants (71% female, 29% male; age range 18–73 years, M age = 35 years, SD = 15), less than half (46%) were married or in a de facto relationship. Most participants had completed technical or vocational degrees, undergraduate or postgraduate level studies (69%), and were employed (69%). Overall, there was a high completion rate of the daily prompt surveys: most participants (75%) responded to at least 35 of the 42 possible prompts (M = 36 prompts; SD = 10), with 50% of participants responding to 40 or more prompts.

Seventy‐six participants were from Australia and 29 from the UK. Australian participants were significantly older (M difference 95% CI: 8.67–17.59 years), reported less overall sleepiness (M difference 95% CI: −0.41 to −0.14), and had weaker behavioural habit strength (M difference 95% CI: −0.33 to −0.07) than UK participants. There were no other detected differences between Australian and UK participants.

As Table 1 shows, at baseline, participants reported generally good‐quality sleep, above the 1–4 scale midpoint (M = 2.85, SD = 0.60). Mean scores of daytime sleepiness were just below the scale midpoint, suggesting that, on average, participants reported feeling rather alert (M = 4.25, SD = 1.9). The average behavioural habit strength rating across the prompts was near the 1–7 automaticity scale mid‐point (M = 4.34, SD = 1.85). The ICCs revealed that both behavioural habit strength and daytime sleepiness had significant within‐person and between‐person variability (Table 1). More than two‐thirds of variability in sleepiness and behavioural habit strength was attributable to moment‐to‐moment changes within‐person.

**TABLE 1:** Demographic statistics of daytime sleepiness and behavioural habit strength.

Variable	*n*	M	SD	Range	ICC (95% CI)
Age	105	34.68	14.83	18–73	—
Gender	105	*%*	—	—	—
Female	75	71.4%	—	—	—
Male	30	28.7%	—	—	—
Other	0	0.0%	—	—	—
Cohort					
Australia	76	72.3%	—	—	—
UK	29	27.6%	—	—	—
Sleep quality	104	2.85	0.60	1–4	—
Sleepiness	3754	4.25	1.90	1–10	0.22 (0.18–0.28)
Habit strength	3755	4.34	1.85	1–7	0.28 (0.22–0.34)

The total number of surveys completed was 3755 by 105 participants.

CI, confidence intervals; ICC, intraclass correlation coefficient values.

### 4.2 Sleepiness and habit strength

Table 2 shows the coefficient estimates and the CIs of the fixed effects from the model testing the associations between sleepiness and behavioural habit strength, controlling for age, gender, cohort and sleep quality. Sleepiness and behavioural habit strength were not associated at the between‐person level; that is, people who reported more overall sleepiness across the study were no more or less likely to engage in strongly habitual behaviours than those who reported less sleepiness overall. Hypothesis 1 was not supported.

**TABLE 2:** Fixed effect estimates of multilevel linear model testing the relations between daytime sleepiness and daily habit strength at between‐ and within‐person levels.

Fixed effects	*b*	SE	95% CI
Intercept	4.20*	0.67	2.92 to 5.49
Between‐person sleepiness	−0.14	0.11	−0.34 to 0.08
Momentary sleepiness	0.14*	0.02	0.11 to 0.17
Sleep quality	0.19	0.17	−0.13 to 0.52
Age	0.00	0.01	−0.01 to 0.02
Gender (male versus female)	0.33	0.23	−0.12 to 0.77
Cohort (Australia versus UK)	0.31	0.25	−0.17 to 0.79

*Statistically significantly different from zero. CI, confidence interval. CIs based on 100 posterior simulations.

**FIGURE 1:**
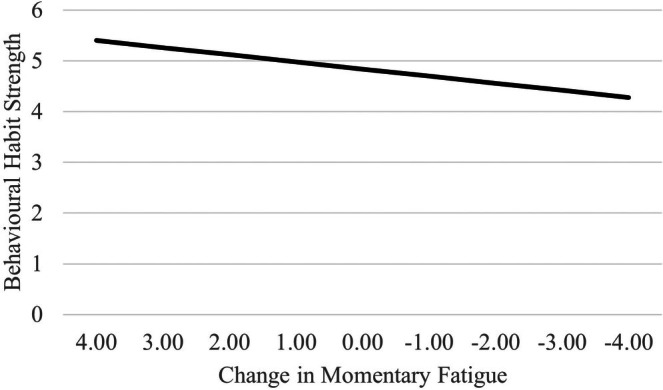
The relationship between behavioural habit strength and change in momentary fatigue.

However, sleepiness had a positive relationship with behavioural habit strength at the within‐person level: participants who were feeling more sleepy than was usual for them were more likely to have been acting habitually. Hypothesis 2 was thus supported. For each single point of fluctuation in momentary sleepiness (on the 1–10 scale), behavioural habit strength was 0.14 units stronger (on the 1–7 habit strength scale), for a SD change of +0.09 (See Figure 1). For effect size estimation, Pseudo‐*R* ^2^ analyses revealed that the within‐person fluctuations in daytime sleepiness explained 2.0% of variability in behavioural habit strength (Δ log likelihoods = 72.97, *df* = 1, *p* < 0.01). Behavioural habit strength was not associated with sleep quality or age. There were no gender or cohort differences in behavioural habit strength.

### 4.3 Post‐hoc exploratory analyses

To explore temporal directionality of the association between sleepiness and behavioural habit strength, two post‐hoc models were estimated with lead and lagged variables. The first model was the same as presented above (between‐person and the concurrent within‐person sleepiness, age, gender, cohort and sleep quality regressed onto behavioural habit strength) with the exception of the addition of a lagged within‐person sleepiness variable (sleepiness of the next momentary assessment for each individual). The results showed no change in the model results and no significant lead effect (95% CI: −0.03 to 0.03), suggesting that behavioural habit strength was associated with concurrently measured sleepiness but did not have an additional effect on later sleepiness. Next, a model was estimated with sleepiness regressed onto behavioural habit strength at the between‐level, as well as concurrently and lagged at the within‐person level (with the same covariates as the previous models). This model revealed that the association remained at the within‐person level concurrently (95% CI: 0.12–0.19) but not lagged (95% CI: −0.01 to 0.06).

## 5 DISCUSSION

This study constitutes the first ecological momentary assessment investigation of the relationship between daytime sleepiness and the habitual nature of behaviour. Both daytime sleepiness and behavioural habit strength were found to vary substantially across the week. Most variability in both sleepiness and behavioural habit strength was at the within‐person level, suggesting that sleepiness and behavioural motivation are dynamic and should be studied using repeated measures designs. Whether people were overall more or less sleepy *than others* had no association with how habitual their daily behaviours were. However, when people were sleepier *than was usual for them*, they tended to be engaging in more strongly habitual behaviours. Post‐hoc analyses revealed that these effects were only for concurrent sleepiness and behavioural habit strength: whether someone was engaging in a strongly habitual behaviour or not did not have an impact on how sleepy they would be later on, and how sleepy a person was in a moment did not have an impact on whether they engaged in more or less habitual behaviours or not later on. These findings align with the theory that the self‐regulatory capacity needed to oppose the automatic influence of habits on behaviour can be deprived (at least momentarily) with sleepiness.

Our participants tended to report engaging in more strongly habitual behaviour when they were sleepier than usual, although the effect was small and only for concurrent measurements of behavioural habit strength and sleepiness. This suggests that during instances of sleepiness, people may slightly favour acting automatically, in line with learned associations, rather than mindfully and deliberatively. Our findings align with the theoretical proposition that sleep is a self‐regulatory resource, the reduction of which may lead to more automatic behaviours that are less reliant on self‐control (Christian & Ellis, 2011; Pilcher et al., 2015). Previous studies have supported this theory, with individuals opting for less challenging tasks when sleep‐deprived than when fully rested (Engle‐Friedman et al., 2010; Engle‐Friedman & Riela, 2004). Likewise, multilevel analyses of diary entries across 5 days from 154 employees revealed that employees procrastinated more on days following worse sleep quality, with this effect being amplified when they also suffered social sleep lag (Kühnel et al., 2016).

Our findings also suggest that, despite sleepiness modestly compromising self‐regulatory capabilities, people can continue to engage in wanted behaviours where those behaviours are habitual. This echoes a body of research showing that making a wanted behaviour habitual can shield that behaviour against the potentially deleterious effects of sleepiness, sustaining engagement in that behaviour despite a momentary lack of self‐regulation (Neal et al., 2013). That sleepiness and behavioural habit strength fluctuated so much across a week within‐individuals suggests they are quick‐to‐change and are potential targets for intervention. Amidst other factors that impact cognitive load, sleepiness should be considered when tailoring the timing and content of behaviour change interventions (Millar, 2017; Schneider et al., 2023). Intervention developers should consider promoting habit formation for desirable behaviours that should be performed in situations when people are likely to be sleepier than normal, such as minimally stimulating (i.e. boring) environments, or during early mornings or late evenings (Bouwman et al., 2022; Millar, 2017). For example, oral health practices such as flossing one's teeth are typically recommended in the morning upon waking, or in the evening before going to bed (Judah et al., 2013), at both of which times self‐regulatory capacity may be reduced due to sleepiness (Åkerstedt et al., 2013). Effective habit formation interventions should in theory increase the frequency and consistency with which such behaviours are done despite sleepiness.

A complementary intervention strategy might focus on modifying unwanted, “bad” habits that people engage in when sleepy, and that conflict with and so hinder the pursuit of desired, “good” behaviours. For example, evidence suggests that people who sleep for shorter periods tend to show more irregular eating patterns, and eat higher‐calorie foods, at least partly due to self‐regulatory failure, which may undermine attempts to maintain positive weight‐management behaviours (Dashti et al., 2015; Gardner et al., 2021). However, factors that impact cognitive load, including sleepiness, pose an important challenge to interventions that seek to make or break habits: sleepiness may render people slightly less able to summon the willpower to repeatedly enact new behaviours, or inhibit old habits, so that new habits can form. Interventions seeking to make or break habits in settings in which people are sleepier than usual should combine habit‐based advice with self‐regulatory strategies, including action planning, monitoring and reminders, until “good” habits are fully established or “bad” habits are discontinued (Quinn et al., 2010; Rebar et al., 2023). Finally, for interventions that aim to support behaviours that must be enacted mindfully, intervention developers should promote engagement in settings, or at times of day, when people are less likely to be sleepy, and so have the self‐regulatory capacity to act non‐habitually.

While within‐person variation in sleepiness affected engagement in habitual behaviour, we found no relationship of between‐participant sleepiness and habitual behaviour; people scoring more highly on trait sleepiness were no more likely to be engaging in a habitual behaviour than others. Additionally, the post‐hoc analyses suggest the linking of sleepiness with behavioural habitualness is present only for concurrent sleepiness and behavioural habit strength. These findings highlight the dynamics of these constructs and echo previous research showing that variation in ego depletion – i.e. impaired self‐regulation caused by prior over‐exertion of self‐regulatory resources – can impact behavioural decision‐making, but only to the extent that it fluctuates within a person over the course of a day (Rebar, Dimmock, et al., 2018). Specifically, when people were more ego depleted than was normal for them, they reported intending to exercise for less time the next day than when they were less ego depleted, suggesting that ego depletion reduced the immediate inclination to engage in physically effortful behaviour (Rebar, Dimmock, et al., 2018). There was no difference in decisions between people who were typically more ego‐depleted and all other people (Rebar, Dimmock, et al., 2018). Taken together with previous research, our findings highlight the importance of considering sleepiness and other sources of self‐regulatory capabilities as the product of both individual differences, which vary between people, and fluctuations within a person over time, and separating out these influences as potential predictors of momentary motivational states (De Valck & Cluydts, 2003).

We used an intensive data collection method over several days with a large cross‐national sample to examine daytime sleepiness and habit strength in everyday life. Frequent, real‐time collection of data from a diverse sample allowed for analysis of within‐person and between‐person variance in sleepiness and habit strength across a week, likely applicable to everyday life within the studied populations. The strengths of the study design and concordance of the present findings with existing research and theoretical models bolster our confidence in the conclusions drawn; however, limitations must be considered.

Momentary sleepiness is only one factor that impacts cognitive load. It is worth investigating the impact of other potential cognitive loads, such as perceived stress and executive functioning, on the likelihood of engaging in habitual behaviour. Likewise, habit is only one source of input into behaviour (Gardner et al., 2024). Any effect of momentary sleepiness on other motivational processes such as self‐regulation and intention implementation would also be impactful for health behaviour interventions. Although our aim was to observe naturally occurring motivation and behavioural processes, participants may have been sensitive to the monitoring of their behaviour, leading to biases in reporting, or changes in motivation or behaviour. More indirect, less intrusive measurement of habits in daily life are needed. Additionally, the study needs to be replicated in a more heterogenous sample, given the high education levels and limited ethnic diversity of our sample. Studying populations who are more prone to waketime sleepiness (e.g. those with sleep disorders, shiftworkers, clinical populations such as those with sleep disorders) may also clarify any relationships between sleep disturbances and habit strength, in turn informing interventions to improve sleep health and everyday functioning.

### 5.1 Conclusion

Our study showed that when people were sleepier than was usual for them, they were slightly more likely to be engaging in more habitual behaviour than when they were less sleepy. We recommend that intervention developers seeking to promote desirable, “good” behaviours that are best performed in conditions conducive to sleepiness, focus on making such behaviours habitual, and dismantling any competing “bad” habits in such settings, to ensure continued and consistent performance. Interventions to support performance of cognitively effortful behaviours might fruitfully focus on enactment in settings in which people are least sleepy, to ensure people have the self‐regulatory resources needed to enact such behaviours effectively.

## AUTHOR CONTRIBUTIONS

**Theresa McLaurin:** Conceptualization; investigation; writing – original draft; writing – review and editing; formal analysis. **Benjamin Gardner:** Conceptualization; investigation; writing – original draft; methodology; writing – review and editing; supervision. **Alexandra E. Shriane:** Conceptualization; investigation; methodology; writing – review and editing; project administration. **Amanda L. Rebar:** Conceptualization; investigation; writing – original draft; methodology; writing – review and editing; formal analysis; supervision; project administration. **Grace E. Vincent:** Conceptualization; investigation; writing – original draft; methodology; writing – review and editing; formal analysis; supervision.

## CONFLICT OF INTEREST STATEMENT

The authors have no non‐financial or financial conflicts of interest to declare.

## PATIENT CONSENT STATEMENT

All participants provided informed consent prior to participating in the study.

## Data Availability

The data that support the findings of this study are openly available in Open Science Framework at https://osf.io/bepj4/?view_only=bfee51ccb52a46eca28ab954bf41464e.
